# Limited professional guidance and literature are available to guide the safe use of neuromuscular block in infants

**DOI:** 10.1111/apa.12682

**Published:** 2014-06-20

**Authors:** Maik Honsel, Cristina Giugni, Joe Brierley

**Affiliations:** Paediatric and Neonatal Intensive Care Unit, Great Ormond Street Hospital for Children NHS Foundation TrustLondon, UK

**Keywords:** Critical care, Neonatology, Neuromuscular blockade, Neuromuscular monitoring, Pharmacology

## Abstract

**Aim:**

Neuromuscular blocking agents (NMBAs) are used in a range of critical illnesses in neonates and infants, despite a lack of guidelines and professional standards. This study reviewed the current evidence base and ascertained UK practice regarding the continuous use of these agents in this age range.

**Methods:**

We reviewed the literature and carried out a telephone questionnaire of all tertiary units in England and specialist children's hospital neonatal units in the UK.

**Results:**

No best practice guidelines or general consensus statements were found, and the only randomised trial to feature an NMBA protocol expressed concerns about its use in such young babies. Of the 56 units contacted, 54 (96.4%) shared information. Only three of the 56 (5.4%) used intermittent boluses of NMBAs, 91.1% used NMBA infusions, 11 (19.6%) routinely used regular neuromuscular blocker pause to assess depth, and only one (1.8%) used peripheral nerve stimulation monitoring. All the units carried out clinical assessments, but only one (1.8%) had a written protocol.

**Conclusion:**

There is a paucity of literature and professional standards to guide the safe use of NMBAs in infants. Of the 54 units who participated in the survey, only one had a protocol for using NMBAs in babies.

## Key notes

- Neuromuscular blocking agents (NMBA) are used in a range of critical illnesses in neonates and infants, despite a lack of guidelines and professional standards.
- Our study found a paucity of literature and professional standards to guide the safe use of NMBAs in infants.
- Of the 54 (96.4%) specialist UK units who took part in our telephone questionnaire survey, only one had a protocol for using NMBAs in babies.

## Introduction

The development of the neuromuscular junction in humans starts as early as 8 weeks of gestation, with the transition from polyneuronal to mononeuronal innervation completed by 25 weeks of gestation 1. For this reason, clinicians use neuromuscular blocking agents (NMBAs), or muscle relaxing drugs, when they intubate neonates 2, to facilitate difficult ventilation and to allow wound healing after surgery when movement would risk anastomotic disruption. NMBAs can be given either by intermittent boluses or by continuous infusion.

Neuromuscular blocking agents can prevent dangerous neonate-ventilator asynchrony, such as in severe meconium aspiration syndrome with persistent pulmonary hypertension or in the presence of an air leak. There is evidence that asynchronous mechanical ventilation in preterm neonates leads to a higher incidence of pneumothorax 3 and higher peak transpulmonary pressures 4 and is likely to increase the risk of intraventricular haemorrhage 5. The development of assisted ventilation modes has helped to decrease such risks, but a small population of neonates will continue to struggle on a ventilator, despite adequate sedation. In these situations, NMBAs can be helpful.

The use of an NMBA is also indicated in other situations, including after surgery, to facilitate wound healing by averting tension on tight anastomosis, for example, after oesophageal atresia or bladder extrophy repair. In cardiac units, NMBAs are used to decrease energy expenditure during low cardiac output, for example after a cardiopulmonary bypass or in myocarditis/cardiomyopathy. They can also facilitate extracorporeal membranous oxygenation (ECMO) in severe pulmonary hypertension or be used when there is delayed sternal closure after bypass surgery. Furthermore, NMBAs are useful during interhospital transfers of intubated neonates, either because of the extent of the baby's illness or the unavailability of assisted ventilation modes.

Neonates, however, have been shown to be relatively sensitive to exposure to NMBAs in comparison to older children. They display proportionately longer action times 6 and therefore require smaller doses 7.

Neuromuscular blocking agent use is associated with complications, including prolonged blockade through over dosage 8, postventilation muscle weakness 9, hypotension 10 and hypoxaemia 11. Nevertheless, the early use of NMBAs in specific conditions has been shown to minimise the side effects 12. Specific complications are known to occur with depolarising NMBAs, such as succinylcholine, including bradycardia 13 and potassium release 14. Inadequate sedation or anaesthesia can lead to awareness during neuromuscular blockade in those undergoing procedures, which was the subject of a national survey of anaesthetic practice in the UK with results currently being awaited 15. Clearly, neonates and infants cannot communicate such awareness, and particular vigilance is necessary.

A structured approach to the safe administration and surveillance of the effects of NMBAs is clearly optimal. However, while consensus statements and accepted standards regarding NMBA use and assessment exist in both adult and paediatric intensive care units 16, there seems to be limited information available to guide those using NMBAs in neonatal intensive care units (NICUs). There are some supportive data regarding short-term efficacy of intermittent NMBA use in neonates, but the long-term effects remain unknown 17. Although first principles suggest that an infusion seems appropriate to provide continuous NMBA, intermittent boluses may be used depending on the indications. In reality, there is little evidence to support the use of continuous NMBA administration in neonates, although this seems to be a commonly used mechanism of delivery. Whatever the mode of administration, close monitoring of efficacy seems warranted. Again, there is little information available on how best to implement this, in particular whether clinical assessment, formal acceleromyography or peripheral nerve stimulation monitoring (PNSM) using a train-of-four monitor is optimal. In our NICU, in a specialist children's hospital, we regularly interrupt the administration of the NMBA infusion to provide the patient with a drug holiday until they shows signs that they are recovering from the drug's effect. PNSM is rarely used in neonates, although electromyography is undertaken for formal neuromuscular assessment.

Because there are so little data available regarding the safe use of NMBAs in neonates, we wanted to ascertain current NICU practice in the UK before further exploring NMBA use.

## Methods

An online literature search for NMBA assessment in neonates was performed using PubMed and NHS Evidence (to Jan 2012), The Cochrane Library (2012), MEDLINE (from 1966 to January 2012), EMBASE (from 1988 to January 2012) and Cinahl (from 2005 to January 2012). The search terms used were neuromuscular block, muscle relaxant AND neonate, infant, NICU, SCBU.

A telephone survey of all tertiary NICUs in England and specialist children's hospital NICUs in the UK was performed during daytime hours in May 2012. The list of English tertiary NICUs was obtained from the National Neonatal Audit Programme (www.rcpch.ac.uk/nnap), and the list of specialist children's NICUs was obtained from the Paediatric Intensive Care Audit Network (http://www.picanet.org.uk/).

We asked the nurse in charge to complete the survey, as we felt this would provide realistic answers about what actually happened in practice, although all were allowed to consult their unit guidelines, if appropriate. A simple four-item questionnaire was deliberately used to minimise disruption to staff (Table1). This covered: whether NMBA protocols existed and were used, whether regular clinical assessments were carried out while using NMBAs, the methods used to assess the effects of NMBA (clinical observations, PNSM, acceleromyography), and the use of drug holidays – intermittent cessations of NMBA infusion – to assess the depth of block, either by clinical movement or by PNSM. We also engaged in a more general discussion about the experience and frequency of NMBA use in the unit, if time allowed.

**Table 1:** Simple four-item questionnaire was used deliberately to minimise staff disruption

Is there a protocol for NMBA use in your unit?
Do you apply PNSM (e.g. train-of-four)?
Do you apply regular clinical assessment while using NMBA?
Do you perform regular drug holidays?

NMBA = Neuromuscular blocking agents; PNSM = Peripheral nerve stimulation monitoring.

## Results

The literature search showed no structured assessment of the depth of NMBA in the 12 publications that were featured in the Cochrane systematic review updated in 2009 17. Six of these studies were randomised controlled trials on the use of muscle relaxants in preterm infants that were published more than 20 years ago.

We found one randomised controlled trial that compared cisatracurium and vecuronium infusions in neonates and small infants after congenital heart surgery and which used routine PNSM to assess the NMBA block 18. In that study, the depth of the NMBA was determined using standard train-of-four monitoring applied over the ulnar nerve and hand. The authors stated that: ‘In nearly all of the neonates, the magnitude of the electromyographic response in the adductor pollicis was too small to be detected by the monitor. Therefore, all determinations of train-of-four response were made by visual observation of thumb movement’ 18.

No clinical practice standards or peer-reviewed guidelines for assessing the effects of NMBA in neonates were found.

We also carried out a telephone survey of 56 units in England and the UK, excluding our own. Most of the staff were happy to reply to our questions, but two units refused to share any information.

Of the 54 (96.4%) units that took part, only three (5.4%) used intermittent NMBA boluses rather than continuous infusions and did not have a PNSM or any other protocol in place. Questions three and four were therefore not relevant (Table1).

The remaining 51 units (91.1%) of the 56 contacted used and were familiar with NMBA infusions. They were used occasionally in tertiary level NICUs and depending on case-mix in the specialist children's hospitals, with frequent use in cases of congenital heart disease, ECMO and pulmonary hypertension.

All the units clinically assessed babies on NMBA, but only one (1.8%) had a written protocol in place.

Regular drug holidays to assess NMBA effect and depth of blockade were routinely performed in 11 (19.6%) units, but only one (1.8%) reported that they would further assess the depth of the blockade by PNSM if a baby did not move after 6 h on a drug holiday.

## Discussion

There are extensive data in the literature, as well as best practice guidelines, regarding the use and monitoring of NMBA in paediatric and adult critical care patients. However, we found no peer-reviewed standards or guidelines in the literature that addressed, or validated, the assessment of neuromuscular blockade in neonates. Patient size limits the application of the standard monitoring devices regularly used in larger children, although anaesthetist colleagues anecdotally describe assessing the depth of NMBA by PNSM of the facial nerve in smaller patients during operations.

The Cochrane database review of neuromuscular paralysis in newborn infants receiving mechanical ventilation was last updated in 2009, but we were unable to find any more recent studies during our literature search. The six randomised controlled trials in the Cochrane review were more than 20 years old, from an era of less sophisticated synchronised ventilation and intensive care in general. None of them featured any NMBA administration and assessment protocol.

We only found one study that included an NMBA protocol and that was a study of infants who were randomised to different NMBA agents after congenital heart surgery. The study expressed concerns about applying standard adult/larger child monitoring techniques to infants 18.

It is, perhaps, surprising that there appears to be such limited clinical and research evidence to guide safe and effective practice for this important group of drugs in such a complex intensive care population. As a consequence, the pharmacodynamics and pharmacokinetics of neonatal NMBA are poorly understood, as indeed are the influence of prematurity and other complicating conditions on the neonatal neuromuscular junction *per se*.

Clinical guidelines and standards to facilitate the consistent and safe use of NMBA in neonates are surprisingly absent. Because of this, the long-term effects of NMBA use in this population are unknown.

It seems reasonable to state that we need to pay greater attention to training neonatal staff in the use of NMBAs and to creating protocols for their safe use and assessment. In general, standardised protocols are particularly helpful for treatment regimens that are either not regularly used or perhaps poorly understood. This creates both confidence in staff and a base for reporting outcomes and further research.

Our telephone survey was well received, with 54 of the 56 (96.4%) units happy to participate, and many suggesting that they would really like guidance to be in place to help their practice. NMBAs are used, albeit infrequently, in tertiary neonatal units, as both intermittent boluses and continuous infusions. However, they are used more frequently in specialist children's hospital NICUs, which care for infants with congenital heart disease, those receiving surgery, including airway surgery, and patients with peri-ECMO pulmonary hypertension.

However, the lack of any validated standards or guidelines is reflected in the fact that most units treating neonatal patients do not have a NMBA protocol for their unit. Clinically assessing the baby is still the main tool used by NICU staff. Arguably, this should happen during drug holiday periods, when the drug was not being administered, but only a fifth of units did this.

## Conclusions

There remains a clear lack of literature regarding the use and assessment of NMBA in neonatal patients. Considering the possible toxicity and side effects of these drugs, further research into their effects on neonatal physiology is needed. In view of this lack of evidence, there is also an urgent need for clinical guidelines and standards for NMBA use in neonates.
